# Generalizability of muscle synergies in isometric force generation versus point-to-point reaching in the human upper extremity workspace

**DOI:** 10.3389/fnhum.2023.1144860

**Published:** 2023-07-17

**Authors:** Katherine Pham, Manuel Portilla-Jiménez, Jinsook Roh

**Affiliations:** Department of Biomedical Engineering, Cullen College of Engineering, University of Houston, Houston, TX, United States

**Keywords:** motor control, muscle synergy, electromyography, isometric force generation, kinematic reaching, human arm workspace

## Abstract

Isometric force generation and kinematic reaching in the upper extremity has been found to be represented by a limited number of muscle synergies, even across task-specific variations. However, the extent of the generalizability of muscle synergies between these two motor tasks within the arm workspace remains unknown. In this study, we recorded electromyographic (EMG) signals from 13 different arm, shoulder, and back muscles of ten healthy individuals while they performed isometric and kinematic center-out target matches to one of 12 equidistant directional targets in the horizontal plane and at each of four starting arm positions. Non-negative matrix factorization was applied to the EMG data to identify the muscle synergies. Five and six muscle synergies were found to represent the isometric force generation and point-to-point reaches. We also found that the number and composition of muscle synergies were conserved across the arm workspace per motor task. Similar tuning directions of muscle synergy activation profiles were observed at different starting arm locations. Between the isometric and kinematic motor tasks, we found that two to four out of five muscle synergies were common in the composition and activation profiles across the starting arm locations. The greater number of muscle synergies that were involved in achieving a target match in the reaching task compared to the isometric task may explain the complexity of neuromotor control in arm reaching movements. Overall, our results may provide further insight into the neuromotor compartmentalization of shared muscle synergies between two different arm motor tasks and can be utilized to assess motor disabilities in individuals with upper limb motor impairments.

## 1. Introduction

Humans and animals can perform a variety of motor tasks in the upper extremity, from motionless force generation to active reaching, as a part of their essential activities of daily living. For instance, isometric force is generated for grasping objects as food against gravity or holding a newborn while nursing. Similarly, reaching behavior is critical to perform most daily activities, such as feeding, transportation, fleeing from predators, and interacting with other species. Both types of motor tasks involve the coordination of the highly complex musculoskeletal system and the central nervous system (CNS). Accordingly, various scientists have studied how the CNS would choose one option of an appropriate muscle activation pattern out of the theoretically infinite number of possibilities to perform a given motor behavior. One simplifying approach is to consider that the CNS reduces the number of degrees of freedom (DOF) to a few muscle synergies, here defined as consistent multiple-muscle coordination patterns that are activated to generate a variety of movements, to achieve a particular motor goal (Tresch and Jarc, 2009; Bizzi and Cheung, 2013; Ting et al., 2015). The term “muscle synergy” has also been used to define stereotypical patterns of muscle activations in the field of neuropathologies, such the stroke flexion synergy (Ellis et al., 2017). However, in this study, we do not define the term “muscle synergy” as such. In addition, a recent study examining the effect of epidural spinal cord stimulation in motor-complete spinal cord injury in muscle synergies found that the number of muscle synergies decreased and the patterns more defined after treatment, further supporting the idea of the neural basis of muscle synergies (Singh et al., 2023). Activation of a few muscle synergies can be an effective strategy by reducing many DOF to generate isometric forces and reaching movements.

Isometric tasks have been studied in motor control from a muscle synergy perspective, and these studies have demonstrated that different isometric motor behaviors can be accomplished by activating a small number of intermuscular patterns in the upper body (Rashedi et al., 2010; Borzelli et al., 2012, 2013; Berger and d’Avella, 2014; Budhota et al., 2017; Ortega-auriol et al., 2019; Roh et al., 2019). For instance, four to five synergies were required to predict upper extremity muscle activation across different spatial, load, and position protocols (Roh et al., 2012), and during visuomotor adaptation (Gentner et al., 2013). Also, a set of three or four trunk synergies could efficiently explain electromyographic (EMG) data during multidirectional target-matching conditions and two different maximum voluntary exertion tasks (Sedaghat-Nejad et al., 2015). Therefore, these studies have shown the robustness of muscle synergies across different isometric conditions in neurologically intact persons.

Previous studies addressing the arm reaching movement have uncovered that the activation of a multitude of muscles could be represented in a minimal number of muscle synergies (d’Avella et al., 2006, 2008; Cheung et al., 2009; Muceli et al., 2010; Coscia et al., 2014; Scano et al., 2019; Valk et al., 2019). More specifically, the activation of the arm, shoulder, and back muscles during multidirectional point-to-point reaching in healthy participants was majorly represented with three to five muscle synergies under different biomechanical conditions, such as changing loads and forearm postures (d’Avella et al., 2006), upper arm orientations (Scano et al., 2019), and end effectors (Valk et al., 2019). A number of muscle synergies were observed to be identical across participants and motor performance conditions (d’Avella et al., 2006; Scano et al., 2019), although distinct task-condition-specific synergy activations were also observed (Valk et al., 2019). The shared muscle synergies under different task conditions suggest that the CNS generalizes the modular organization of muscle activations given the same motor task.

With the knowledge that both isometric force generation and arm reaching can be produced by a small number of muscle synergies, the question then arises on the degree to which muscle synergies between these upper extremity motor tasks are shared. Only a limited number of research studies have investigated the extent of the generalizability of muscle synergies between different motor tasks, especially in the human upper extremity. For instance, a frog study showed that three behavior-independent and two behavior-specific synchronous muscle synergies could explain the invariances during jumping, swimming, and walking (d’Avella et al., 2003; Roh et al., 2011). One study in the lower extremity showed common muscle synergies for balance and walking in humans during perturbation responses (Chvatal and Ting, 2013). A walking versus cycling study (Barroso et al., 2014) showed that cycling synergies could be explained by merging walking synergies in humans. This finding suggests that cycling and walking share common neuromuscular mechanisms. Overall, these studies provided evidence for a common set of muscle synergies used for different motor behaviors, more specifically in animals and the human lower extremity, which could indicate that the CNS elicited the same activation of complementary muscle groups across a variety of movements. However, it is still unclear if a set of muscle synergies could be similarly compartmentalized in the upper extremity across different motor tasks, including isometric and kinematic reaching.

Thus, the aim of this study was to investigate the degree to which muscle synergies were generalized between isometric force generation and kinematic reaching across the human arm workspace within the horizontal plane. We hypothesized that muscle synergies would be robust and generalizable between the isometric force generation and point-to-point reaching tasks across different starting arm locations in the human arm workspace. Ten young, healthy participants performed the same horizontal center-out target matches under two biomechanical conditions, one while the arm remained stationary and the other with kinematic movement, while surface EMG signals, end-point force at the hand, and reaching trajectory were recorded. The composition and activation profiles of muscle synergies, identified by non-negative matrix factorization (NNMF), were used in analyzing the two motor tasks across target directions and starting arm locations.

## 2. Materials and methods

### 2.1. Participants

Ten young, able-bodied participants [age: 21.64 ± 1.91 years (mean ± standard deviation (SD)], 19–26 years (range); five females) were recruited for this study. Participants were included in the study if they were: (1) between the ages of 18 and 40, (2) predominantly right-handed, and (3) able to understand and provide informed consent. The exclusion criteria included: (1) if the participant had neurological impairments that could affect cognition and communication and (2) orthopedic impairments that could affect upper limb motor tasks. This study was performed following the experimental protocol approved by the Institutional Review Board of the University of Houston. All participants provided informed consent prior to the beginning of the study.

### 2.2. Equipment

**FIGURE 1:**
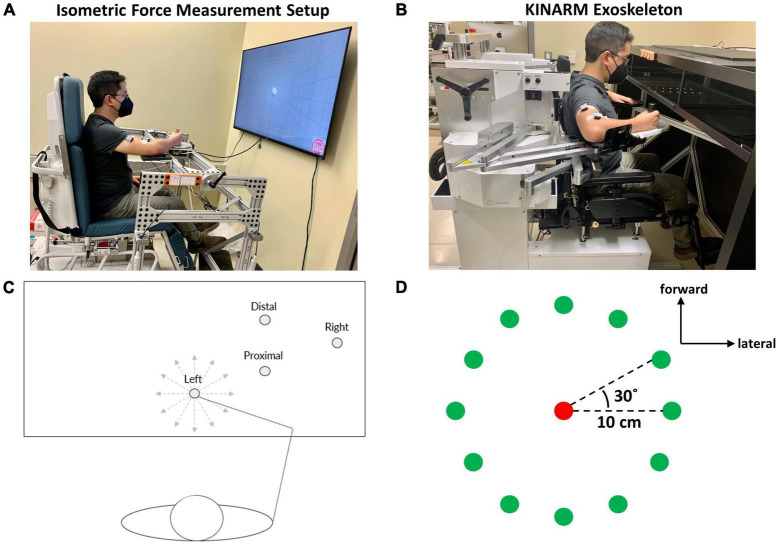
Experimental set-up and protocol for isometric and kinematic reaching tasks performed at one of four starting arm locations. **(A)** The isometric force measurement set-up consisted of a handle for grasping and a force transducer attached to the mechanical frame that enabled the experimental protocol to cover the workspace of the arm in 3D space. During isometric reaching, participants performed visually guided force generation to match force targets while the arm remained stationary. **(B)** The KINARM Exoskeleton used for the point-to-point reaching task. When the participant performed a visually guided point-to-point reach to a virtual target, the device measured the arm trajectory and hand path within the horizontal plane. **(C)** The four different starting arm locations in the human arm workspace for both isometric kinematic reaching tasks. The starting arm coordinates are presented in Section “2.3. Experimental protocol”. The 12 gray, dotted lines indicate the directions of the 12 isometric or kinematic reaching targets. **(D)** The 12 targets (green circles) for center-out isometric and kinematic target matching were equally distributed around a circumference whose center was indicated as a red circle, 30° away from the adjacent target. In the kinematic reaching task, the reaching distance was 10 cm.

The isometric force measurement (IFM) device was a 3D force/torque measurement system that allowed participants to perform upper arm isometric force target matches (Figure 1A). To measure the changes in end-point force during the motor task, a force transducer (Model: 45E15A4, JR3, Woodland, CA) was attached with an extended grasping handle and recorded the 3D applied end-point forces throughout the experiment. A visual target was displayed on a computer screen in front of the set-up. Electromyographic (EMG) and 3D force data were simultaneously recorded using a custom-designed program using the LabVIEW software (National Instruments, TX, USA). The kinetic data was collected at 20 Hz.

The Kinesiological Instrument for Normal and Altered Reaching Movement (KINARM) robotic exoskeleton (BKIN Technologies, Ontario, Canada) was a bimanual robotic system that allowed gravity-supported upper arm movement in the horizontal plane while measuring kinematic data from changes in the shoulder and elbow joints during the performed motor task (Figure 1B). EMG data were also recorded simultaneously. The robot arm was modified with the attachment of the grasping handle to allow a participant to perform point-to-point reaching tasks in the arm configuration used for isometric force target matches. An augmented reality screen was used to visualize a target for a reach. The kinematic data acquisition rate was 1 KHz.

The surface EMG signals (Delsys Trigno Sensors, Delsys, MA, USA) were recorded from 13 different arm/back muscles: brachioradialis (BRD), biceps (BI), brachialis (BRCH), triceps (long and lateral) (TriLong and TriLat), deltoids (anterior, middle, and posterior; AD, MD, and PD), pectoralis (clavicular fibers; PECT), trapezius (upper, middle, and lower) (UT, MT, and LT), and infraspinatus (IN). The electrodes were placed following the SENIAM protocol (Hermens et al., 1999), excluding BRD, BRCH, UT, MT, LT, and IN muscles. For placing EMG sensors on those muscles, the Anatomical Guide for the Electromyographer (Perotto et al., 2004) was also used as a reference. The EMG data acquisition rate was 1 kHz.

### 2.3. Experimental protocol

The study was comprised of two different motor tasks (isometric force generation and point-to-point reaching) (Table 1). The four different arm locations were determined by the global coordinates (x, y) (in cm), based on the maximum active range of motion available in the horizontal plane of the KINARM device. The origin of the global coordinates (0, 0) was the midpoint between the shoulders. The four different starting locations of the hand in the arm workspace were: (1) the Right at (39 ± 0, 47.25 ± 0.98) (mean ± SD, *n* = 10), (2) the Left at (0 ± 0, 40 ± 0), (3) the Proximal at (20 ± 0, 43.75 ± 0.63), and (4) the Distal at (20 ± 0, 52.7 ± 0.67) (Figure 1C). The locations of the starting arm positions were adjusted based on the maximum size of each participant’s arm workspace covered by the task within the mechanical constraints of the KINARM Exoskeleton for reaching. The order of the motor tasks, starting arm locations, and target-reaching direction were randomized.

**TABLE 1:** The summary of the distinction between the isometric force generation task versus the point-to-point reaching task.

Isometric force generation	Point-to-point reaching
● *Equipment:* Isometric Force Measurement Device ● *Motor task:* Participant holds handle and applies force in the horizontal plane without moving the arm to achieve target match ● *Collected data:* Three-dimensional force and EMG data collected simultaneously	● *Equipment:* KINARM Exoskeleton ● *Motor task:* Participant holds handle and moves the arm in the horizontal plane to achieve target match ● *Collected data:* Kinematic and EMG data collected simultaneously
Differences in sensorimotor feedback between motor tasks	

To ensure that the two motor tasks were comparable for further analysis, the posture and the arm position of the participant were kept consistent between the two motor tasks by utilizing the global coordinate system of the KINARM Exoskeleton. Before starting the experiment, the participants were first placed in the KINARM Exoskeleton to measure the arm and elbow angles as well as the distances between the participant’s acromion to the handle at each starting arm location. Before each motor task and each starting arm location, the posture and arm locations were replicated and validated using the recorded measurements.

In the isometric force generation motor task, participants were seated within the IFM system with an arm support tray, to support the weight of the arm, while their dominant hand held onto the grasping handle in front of them. The participant’s non-dominant arm was placed on their lap. The participant’s shoulder was abducted to 80^°^. The participant’s torso was also fixed using a seatbelt to ensure that the participant remained in the same posture for the entirety of the task. To calibrate the system for the experiment, the participant was instructed to relax completely while grasping the handle. The weight of the arm applied onto the device was then zeroed to correctly map and measure the 3D forces during the force generation task. Within the system, *Fx* was defined as the lateral (+*Fx*) and medial (-*Fx*) direction, *Fy* was defined as the forward (+*Fy*) and backward (-*Fy*) direction, and *Fz* was defined as the upward and downward direction. 40% of the maximum lateral force in the +*Fx* direction was measured to determine the force load applied for the target matching task. Since the maximum lateral force was typically the smallest among maximum voluntary contractions in 3D force space, the procedure ensured that a participant could produce the force magnitude in any target force direction. For each starting hand location, the participant performed a force-generated center-out, point-to-point target matching task to one of 12 targets in the horizontal plane, equally distributed on the circumference of a circle, with five repetitions in each direction (Figure 1D). Before the start of the experiment, the participants were instructed to be completely relaxed but mentally ready during the baseline period and to match a force target as fast and as accurately as possible once the target appeared on display. The participants were also instructed not to produce force in the *Fz* direction when performing the target matches in the horizontal plane. Although we collected three-dimensional data in the isometric force generation task, the participants were instructed and monitored to ensure that the isometric force was applied in the horizontal plane only to achieve a target match, comparable to the reaching task. In each trial, the first five seconds were provided as a baseline period to ensure that a stable baseline was measured for further EMG processing per trial, followed by a center-out task with a three-second intertrial interval. The criteria for a successful trial were if the participant was able to remain in the given target area with a logical radius of 20% for one second. Three attempts were given to allow the participant to match the target per trial.

In the point-to-point reaching task, the participant was seated in the chair of the KINARM device and placed their dominant arm into the plastic tray to support the weight of the arm, while their hand held onto the grasping handle at the end of the robotic arm. The participant’s non-dominant arm was placed on their lap. The participant’s shoulder was abducted to 80°. The participant’s torso was also fixed using a seatbelt to ensure that the participant remained in the same posture for the entirety of the task. Then, the participant was placed in front of an augmented reality screen, where the participant could see their arm as well as the visual targets to perform the motor task. The augmented reality screen is a mirror that displays the reflected computer display located above the device and allows the participant to visualize the reaching targets and the participant’s arm movements, in the form of a cursor on the screen, simultaneously (Figure 1B; Grohs et al., 2021; Lowrey et al., 2022). Before the start of the experiment, a calibration process was required to synchronize the robotic device to the computer system (KINARM Lab Operator Guide, BKIN Technologies). For each starting hand position, the participant performed a horizontal center-out, point-to-point reaching task to one of the 12 targets, equally distributed on the circumference of the circle with a radius of 10 cm, with five repetitions in each direction (Figure 1D). In each trial, the first second was the baseline period, and then a center-out reaching task should be performed within one second. The participants were instructed to be completely relaxed and still until the outer target appeared and to reach the target as fast and accurately as possible within 0.2 and 0.35 s. If the criteria were not met, visual feedback of “TOO FAST” or “TOO SLOW” was provided for the participant. The velocity constraint was applied to the task to ensure that the movement-dependent EMG signals were collected consistently for equal comparison across all task conditions and participants. Three attempts at maximum were allowed for the participant to match a target per trial.

### 2.4. Data analysis

#### 2.4.1. EMG processing

All data analysis was done using MATLAB software. The order of the EMG processing was the following: (1) wavelet-based electrocardiogram (ECG) filtering, (2) subtraction of the mean EMG of each muscle to remove the DC offset, (3) full-wave rectification of EMG signals, (4) subtraction of the mean baseline EMG to remove baseline noise, and (5) the 4th order Butterworth low-pass filtering with a cutoff frequency at 10 Hz to extract the EMG envelope.

The end-point force onset and offset were determined by finding where the applied end-point force exceeded and fell below three times the standard deviation of the force baseline from the mean value, respectively (Hodges and Bui, 1996). The reaching trials were trimmed to the movement onset and offset, which was determined by finding where the movement speed of the hand exceeded 10% of the maximum velocity and decreased to 10% of the maximum velocity, respectively (Russo et al., 2014). The trials were then interpolated to 150 samples per trial, which ensured the equal contribution of each trial to identify muscle synergies in the next step. The EMG data were concatenated across trials per each condition to identify muscle synergies. The EMG of each muscle was normalized by the variance of the same muscle to avoid any bias toward muscles with high variance in muscle synergy extraction. The EMG datasets were separated by motor task and starting locations for further comparison in muscle synergy analysis.

#### 2.4.2. Synergy identification and comparison

To perform muscle synergy identification, NNMF (Lee and Seung, 1999, 2001) was applied to EMG matrices from both motor tasks. The EMG matrices were linearly decomposed to a matrix of muscle synergy vectors, or W, and their respective activation profiles, or C, identified from 13 arm muscles as the following


(1) E⁢M⁢G=W⋅C.

To estimate the minimum number of muscle synergies that could represent each dataset (per motor task and per starting arm location), we calculated the variance accounted for (VAF) for each condition as


(2) $$V\,A\,F=100\times \left(1-\frac{S\,S\,E}{S\,S\,T}\right),$$

where SSE was the sum of the squared residuals and SST was the sum of the squared EMG data (Roh et al., 2015, 2019).

The criteria to estimate the least number of muscle synergies needed to reconstruct the EMG data were that the number of synergies should have a mean global VAF (gVAF) > 90%, a difference of global VAF would be less than 5% with the addition of another muscle synergy, and each muscle would have a VAF > 60% (Seo et al., 2022). Based on these criteria, we determined the appropriate number of muscle synergies for each condition.

To compare the synergies of motor tasks and the starting arm locations, we analyzed the global VAF, the muscle synergy composition, and the muscle synergy activation tuning curves from all datasets. The comparison of the muscle synergy tuning curves across the starting arm locations in each motor task was assessed using the local coordinate system. With the local coordinate system, the directionality of the tuning curves was more comparable across the starting arm locations due to changing direction of the arm’s biomechanical action, such as the forward and backward motor directions, at different starting locations (Supplementary Figure 1). The Left starting location was rotated clockwise 28°, and the Right starting location was rotated counterclockwise 22.4°. By using the local coordinate system, we were able to align the biomechanical actions of the arm at different starting locations for the comparison of muscle synergy activation profiles.

#### 2.4.3. Quantifying similarity between muscle synergies

The similarity of muscle synergies between isometric versus kinematic reaching each starting arm location was done by calculating the scalar product of the best matching pairs of muscle synergy compositions out of all possible muscle synergy pairing combinations (Cheung et al., 2005). First, the scalar product was calculated between each muscle synergy across all subjects in the isometric reaching task to each muscle synergy in the kinematic reaching task. The scalar product of all possible pairs of muscle synergies between the isometric and kinematic reaching tasks was accounted for with the removal of the previous pair. The muscle synergy composition was matched between the two tasks for comparison based on the highest sum of similarity of each muscle synergy pair. Then, we calculated the similarity score between the motor tasks by finding the scalar product (*r*) of the best-matched pairs of muscle synergies and its statistical significance, *r* > 0.738, or when *r* surpassed the statistical significance threshold. The threshold was determined by generating 1,000 random sets of muscle synergies whose muscle weights were randomly chosen from the weights of computed muscle synergies that underlay all motor tasks across all arm locations and participants. The scalar products of any potential pairs of the random synergies (*_1000_C_2_* = 499,500) were ordered in an ascending way, and the statistical threshold was determined to be the 95th percentile of the calculated scalar products (*p* < 0.05; Roh et al., 2012, 2013). Based on this method, the 95th percentile of the calculated scalar product resulted in a value within the tenths of 0.738. Thus, the value of 0.738 was selected as the threshold value.

To compare the similarity of muscle synergy composition between the two motor tasks, we calculated the correlation coefficient (*r*) by computing the scalar product of a pair of muscle synergies in comparison. The correlation coefficient value was deemed statistically significant when *r* > 0.738.

To compare the similarity of muscle synergy composition between the different starting arm locations in comparison, we calculated the correlation coefficient (*r*) by computing the scalar product of a pair of muscle synergies in comparison. The threshold was then calculated using the same method above with the Bonferroni correction (α = 0.05/4) to account for the comparison of multiple different starting arm locations. The correlation coefficient value was deemed statistically significant when *r* > 0.837.

In addition to the similarity score analysis, we used a cross-validation method to further analyze the similarity of synergies as a group between the starting arm locations. In this method, we reconstructed the original EMG signals (EMG A) at each location using the other muscle synergies (synergy set B) of all the possible combinations of locations simultaneously, different to comparing the muscle synergies individually between two locations in comparison. To do so, for each pair of all the possible combinations of starting arm locations within the same motor task, we calculated the dot product of the muscle synergy vectors, or W, of the first location (W_1) and the synergy activation profiles, or C, of the second location (C_2) to make a reconstructed EMG of the second starting arm location per participant (EMG_2). Then, for each pair of all the possible combinations of starting arm locations, we calculated the dot product of the muscle synergy vectors, or W (e.g., W_Right), and the optimized synergy activation profiles, or C’ (e.g., C’_Left), of each possible combination of starting arm locations to make reconstructed EMGs (EMG_Left). Then, the gVAF of the reconstructed EMGs for each of the possible pairs of starting arm locations was compared to the gVAF of the original EMG using the two one-sided test for equivalence (TOST) (McGrath and Meyer, 2006; Lakens, 2013, 2017). If the difference between the gVAF value of the reconstructed EMG and the gVAF value of the original EMG falls within the range of 5%, the mean gVAFs between the two groups will be interpreted to be similar. Since our synergy identification criteria requires the difference in gVAF when an additional muscle synergy is added to be within 5%, we used the same criteria to compare the similarity of the gVAF between the two sets of EMGs in comparison in the same manner (TOST, range 5%).

#### 2.4.4. Quantifying the number of synergies activated per target direction

To determine the number of muscle synergies activated per target direction, the magnitude of extracted synergy activation profiles had to exceed the muscle synergy activation threshold. The threshold (*t*) was determined by computing the value in which a statistical property (variance) changed abruptly using the change point detection method (MATLAB function “findchangepts”) (Killick et al., 2012; Truong et al., 2020). First, we combined the muscle synergy activation profiles across all muscle synergies, trials, and participants at each starting location of the same motor task. Then, we sorted the magnitude of synergy activation profiles from all trials in ascending order at each starting location of the same motor task. We applied the change point detection method to obtain this threshold per condition.

#### 2.4.5. Additional statistical tests

The two-sided t-test was performed to evaluate the statistical differences of the number of muscle synergies computed between the two motor tasks per starting arm location.

The single factor ANOVA (α = 0.05) (MATLAB) was used to evaluate the group differences in the muscle synergy composition across different starting arm locations and motor tasks, respectively. Three sets of muscle synergies were computed as three templates (or models) for the isometric task, the reaching task, and both tasks combined, respectively. The models were calculated by obtaining the mean value of all participants and locations (distal, right, left, and proximal) per muscle synergy and transformed into a single vector. The dot product between each participant’s synergy and their respective model synergy was obtained, and the ANOVA across each task and both tasks were performed, respectively. In addition, the tuning directions, which are the angles of the linear summation of all components per each participant’s tuning curve, were obtained to evaluate the group’s differences in synergy activation profiles. A single factor ANOVA (α = 0.05) test for circular data was applied for each task. Finally, to determine which tuning directions were statistically different, the two-sample circular test with Bonferroni correction was performed.

## 3. Results

### 3.1. EMG profiles of the isometric force generation vs. point-to-point reaching

**FIGURE 2:**
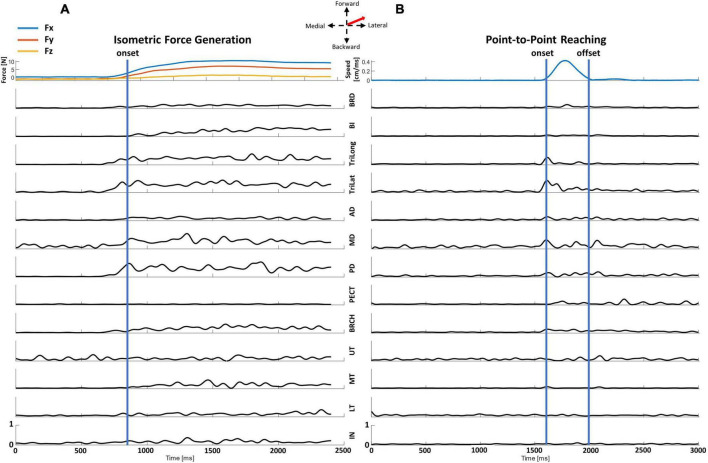
Representative data recorded from a representative participant of a center-out target match trial in the isometric force generation and point-to-point reaching tasks at the Proximal starting location to the same forward-lateral direction. The EMG signal of each muscle was normalized to the maximum muscle contraction of all trials at each motor task to visualize the activation characteristics of each muscle throughout the trial. **(A)** The end-point forces and EMG data of an isometric force target match. The EMG data for each trial was trimmed from the calculated force onset (blue line) to the end of the trial. **(B)** The hand speed and EMG of a point-to-point kinematic reach. The EMG data were trimmed from the calculated movement onset to movement offset (blue lines) for further synergy analysis. Arm and back muscles: brachioradialis (BRD), biceps (BI), brachialis (BRCH), triceps (long and lateral) (TriLong and TriLat), deltoids (anterior, middle, and posterior; AD, MD, and PD), pectoralis (clavicular fibers; PECT), trapezius (upper, middle, and lower) (UT, MT, and LT), and infraspinatus (IN).

The EMG profiles of the two motor tasks (Figure 2) recorded from the identical representative participant to the same target direction at the same starting arm posture were distinct at each trial. From both motor tasks, the elbow extensor (TriLong and TriLat) muscles and the deltoids (mostly MD and PD) were activated, observed as higher magnitudes in the EMG signals, to achieve a target match in the forward-lateral direction. The isometric force generation trial (Figure 2B) had supplementary activation, seen in the EMG signals of the elbow flexion (BRCH, BI, and BRD) and back (UT, MT, LT, and IN) muscles, to support the participant in producing and holding the required directional force for the target match. Across time, the EMG profiles of individual muscles differed in magnitude in the two trials. Individual muscles were activated in the isometric task (Figure 2A) during the force onset and the one-second hold period to achieve the target. The activation of the muscles in the point-to-point kinematic reaching task (Figure 2B) was observed within a shorter duration, less than one second, between the time of movement onset and offset for a target match.

### 3.2. Number of muscle synergies in isometric force generation vs. reaching across starting locations

**FIGURE 3:**
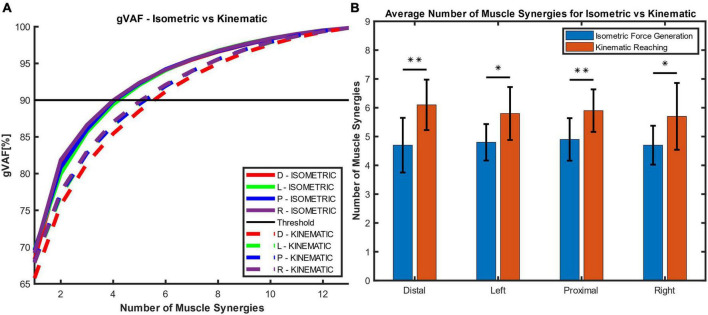
The global variance accounted for (gVAF) and the average number of muscle synergies for each motor task across different starting arm locations. **(A)** The gVAF value of the isometric force generation (ISOMETRIC) and point-to-point kinematic reaching (KINEMATIC) at each starting hand location [distal (D), left (L), proximal (P), right (R)]. The gVAF threshold for the ideal number of synergies was 90%. **(B)** The number of muscle synergies (*n* = 10; mean ± SD) across conditions and across participants. Five and six muscle synergies were found to represent the isometric force generation and point-to-point reaching tasks, respectively. The number of muscle synergies was determined using the synergy identification criteria (see Section “2.4.2. Synergy identification and comparison”). There were significant differences in synergy numbers between the isometric and kinematic reaching tasks (two-sample t-test; *p < 0.05; **p < 0.01).

In the isometric force generation task, approximately five muscle synergies represented the task across the different starting locations. In Figure 3A, five muscle synergies could explain over 90% of the total variance of the EMG patterns collected from 13 muscles at each starting location (VAF at the Distal, Left, Proximal, and Right locations = 92.3 ± 1.69%, 92.05 ± 1.61%, 92.27 ± 1.41%, and 92.39 ± 1.44%, respectively; all mean ± SD). Figure 3B shows the average number of muscle synergies between isometric force generation (ISO) versus point-to-point reaching (REACH) across starting arm locations. Based on the synergy identification criteria, 4.7 ± 0.95 synergies, 4.8 ± 0.63, 4.9 ± 0.74 synergies, and 4.7 ± 0.67 synergies (*n* = 10; mean ± SD) were determined to represent isometric force generation at the Distal, Left, Proximal, and Right locations, respectively.

In the point-to-point reaching task, approximately six muscle synergies represented the task across the different starting locations in the arm workspace. In Figure 3A, at six muscle synergies, the global VAF for the reaching task at each starting location exceeded 90% variance of EMG signals (VAF at the Distal, Left, Proximal, and Right locations = 91.17 ± 2.0%, 91.98 ± 1.76%, 91.92 ± 1.72%, and 92.11 ± 1.8%, respectively; all mean ± SD). On average, 6.1 ± 0.88 synergies, 5.8 ± 0.92, 5.9 ± 0.74 synergies, and 5.7 ± 1.16 synergies (*n* = 10; mean ± SD) were determined to represent point-to-point reaching in the Distal, Left, Proximal, and Right locations, respectively (Figure 3B).

Figure 3B shows that the number of muscle synergies was statistically different between the isometric force generation and point-to-point reaching at each starting location (two-sample t-test, *p* at the Distal = 0.003; *p* at the Left = 0.011; *p* at the Proximal = 0.007; and *p* at the Right = 0.03). The numbers of muscle synergies in the isometric force generation and point-to-point reaching, five and six synergies, respectively, were then used to compute the muscle synergy composition and activation profiles for all participants.

### 3.3. Muscle synergies in isometric force generation across different starting locations

#### 3.3.1. Muscle synergy patterns

**FIGURE 4:**
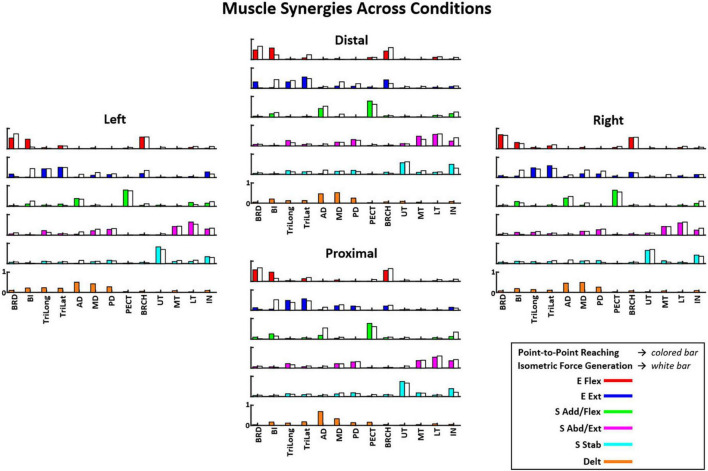
The composition of six muscle synergies (E Flex, elbow flexion; E Ext, elbow extension; S Add/Flex, shoulder adduction and flexion; S Abd/Ext, shoulder abduction and extension; S Stab, shoulder stabilization; and Delt, deltoids) observed during the point-to-point reaching task compared to the five muscle synergies (excluding the Delt synergy) in the isometric force generation task across starting arm locations as mean ± SD (*n* = 10). The muscle synergy composition was consistent with minor variations in the muscle weights across the starting arm locations. The muscle synergy compositions were also majorly similar between the two motor tasks. See the full muscle names of the corresponding acronyms in “2. Materials and methods”, Section “2.2. Equipment”.

Figure 4 shows that the composition of muscle synergies underlying isometric force target matches was similar across the starting arm locations in the upper extremity workspace. Each colored bar represented the mean muscle weight, and the error bar represented the standard deviation from the mean value across all participants (*n* = 10). The five muscle synergies patterns identified in Figure 4 were: elbow flexion (E Flex), elbow extension (E Ext), shoulder adduction and flexion (S Add/Flex), shoulder abduction and extension (S Abd/Ext), and shoulder stabilization (S Stab). The muscle synergies were named following the approximate mechanical action of the muscles activated within each synergy for the isometric force contraction. The E Flex synergy contained the activation of BRD and BRCH muscles, along with some activation of BI and TriLat across starting locations. The E Ext synergy consisted of the activation of the BI muscle grouped with TriLat and TriLong, suggesting that a co-activation of the biceps, a typical elbow flexor muscle, and triceps was involved in stabilizing the elbow during isometric force target matches. Some activation of MD, PD, and BRCH were also observed due to some involvement of shoulder abduction in achieving the motor task. The S Add/Flex synergy involved the co-activation of AD and PECT with some activation of BI and IN. The S Abd/Ext synergy consisted of the activation of the MD, PD, MT, LT, and IN muscles when performing isometric contraction that required the use of back muscles in externally rotating the shoulders. The S Stab synergy involved the lone activation of the UT muscle with some IN, which was involved in mainly stabilizing the shoulder during the motor task.

The muscle synergy composition within the isometric force generation task across the four starting arm locations was not significantly different (ANOVA; E Flex, *F*(3,36) = 1.86, *p* = 0.153, E Ext, *F*(3,36) = 0.68, *p* = 0.57, S Add/Flex, *F*(3,36) = 0.14, *p* = 0.937, S Abd/Ext, *F*(3,36) = 0.67, *p* = 0.574, S Stab, *F*(3,36) = 0.18, *p* = 0.908). In addition, the majority of the synergy similarity scores between any possible pairs of the different starting locations were statistically significant (Table 2; *r* > 0.837), meaning that the composition of most muscle synergies for isometric contraction was conserved in the workspace of the human upper extremity. The muscle synergy with lower similarities was the S Stab synergy, across all starting arm locations. From Figure 4, the S Stab synergy in the Proximal location had a lower IN muscle weight coupled with a higher UT, differing from the other starting locations, which contributed to the lower similarity score in comparison with other arm locations in the workspace. From the cross-validation between a pair of two starting arm locations in comparison in the isometric task, the gVAF values of the reconstructed EMGs were close to the synergy identification gVAF threshold value (90%) and within the range of [87.52, 90.32]. The two one-sided test showed that the differences between the reconstructed EMGs and the original EMG falls within the range of 5% in the isometric task, deeming the mean gVAFs between the two groups equivalent, except for the reconstructed EMG between the right and left starting arm locations and the distal in reconstructing the left starting arm location (Table 3).

**TABLE 2:** The similarity scores of the muscle synergies (mean ± SD; *n* = 10) for isometric force generation between all possible pairs of starting hand locations.

Isometric force generation						
Starting hand location comparisons/Muscle synergy		E Flex	E Ext	S Add/Flex	S Abd/Ext	S Stab
Distal vs. Left	Mean ± SD	0.93 ± 0.04*	0.88 ± 0.09*	0.85 ± 0.21*	0.79 ± 0.22	0.82 ± 0.20
Distal vs. Right		0.96 ± 0.03*	0.85 ± 0.16*	0.93 ± 0.11*	0.85 ± 0.25*	0.80 ± 0.20
Distal vs. Proximal		0.96 ± 0.03*	0.81 ± 0.26	0.84 ± 0.25*	0.88 ± 0.22*	0.68 ± 0.33
Left vs. Proximal		0.95 ± 0.04*	0.88 ± 0.22*	0.85 ± 0.23*	0.87 ± 0.14*	0.66 ± 0.33
Left vs. Right		0.91 ± 0.07*	0.89 ± 0.09*	0.89 ± 0.07*	0.89 ± 0.09*	0.74 ± 0.17
Right vs. Proximal		0.95 ± 0.05*	0.88 ± 0.14*	0.85 ± 0.24*	0.94 ± 0.05*	0.71 ± 0.30

*p < 0.0125. E Flex, elbow flexion; E Ext, elbow extension; S Add/Flex, shoulder adduction and flexion; S Abd/Ext, shoulder abduction and extension; and S Stab, shoulder stabilization.

**TABLE 3:** The gVAF of reconstructed EMGs (mean ± SD; *n* = 10) between a pair of starting arm locations in comparison in isometric force generation using the cross-validation method.

Starting location/Reconstructed EMGs	Isometric force generation			
Distal	**W_D_** × **C_D_**	**W_L_** × **C′_D_**	**W_P_** × **C′_D_**	**W_R_** × **C′_D_**
	92.30 ± 1.69	88.75 ± 2.26*	90.42 ± 2.02***	89.71 ± 2.67*
Left	**W_L_** × **C_L_**	**W_D_** × **C′_L_**	**W_P_** × **C′_L_**	**W_R_** × **C′_L_**
	92.06 ± 1.61	88.47 ± 2.89 (*p* = 0.156)	89.13 ± 1.77**	87.52 ± 2.43 (*p* = 0.312)
Proximal	**W_P_** × **C_P_**	**W_D_** × **C′_P_**	**W_L_** × **C′_P_**	**W_R_** × **C′_P_**
	92.27 ± 1.41	90.32 ± 1.65**	89.49 ± 1.90**	89.83 ± 2.67**
Right	**W_R_** × **C_R_**	**W_D_** × **C′_R_**	**W_L_** × **C′_R_**	**W_P_** × **C′_R_**
	92.39 ± 1.44	89.74 ± 3.34*	88.02 ± 3.01 (*p* = 0.255)	90.03 ± 3.03**

*p < 0.05; **p < 0.01; ***p < 0.001. W, muscle synergies; C, synergy activation profiles; C′, optimized synergy activation profiles; D, distal; L, left; P, proximal; and R, right.

#### 3.3.2. Synergy activation profiles

**FIGURE 5:**
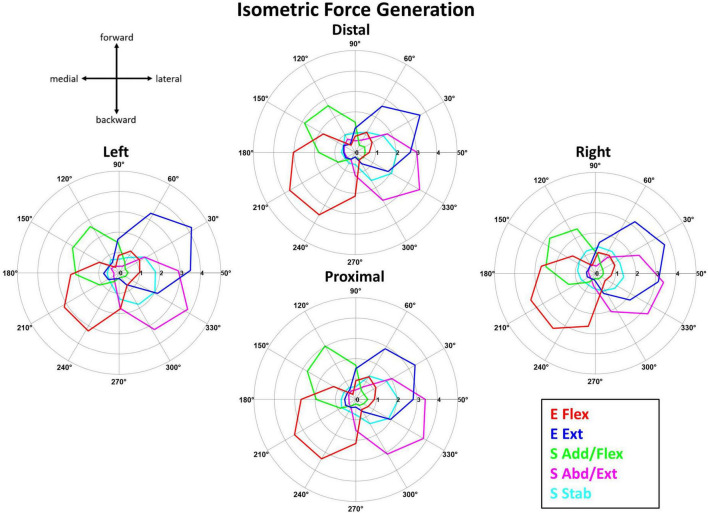
The synergy activation profiles as a function of target force directions in isometric reaching at each of the four starting arm locations. The muscle synergy activation tuning curves showed the averaged contribution (*n* = 10) of each muscle synergy in achieving the isometric force target matches in one of 12 different directions across the four starting arm locations. The starting arm locations were aligned to the biomechanically forward-backward and lateral-medial directions (see Section “2.4.2. Synergy identification and comparison”). The tuning directionality of the synergy activation profiles was consistent (except for S Stab in the Right location), with some differences in the activation magnitudes across the starting arm locations.

Figure 5 shows that the averaged activation profile of each muscle synergy except S Stab was tuned to a distinct subset of the horizontal force space, covered by the 12 force targets. The mean activation profiles (*n* = 10 participants) of the muscle synergies during isometric force generation were plotted at each of the 12 target directions per starting arm location. As mentioned in Section “2.4.2. Synergy identification and comparison”, the starting arm locations were assessed in local coordinates to compare the directionality of the muscle synergy activation. The E Flex and E Ext activation profiles were opposing in their directionality; the E Flex was activated in the backward-medial direction of force generation, while the E Ext was activated in the forward-lateral direction. The S Add/Flex and S Abd/Ext synergies also had opposing activation profiles in the forward-medial and backward-lateral directions, respectively. On the contrary, the activation of S Stab synergy did not have a tuning directionality, notably in the Right location where the activation was centralized across all target directions.

The tuning directions within the isometric force generation task across the four locations was significantly different (*p* < 0.05) only for S Stab synergy [ANOVA; E Flex, *F*(3,36) = 1.41, *p* = 0.255, E Ext, *F*(3,36) = 0.46, *p* = 0.712, S Add/Flex, *F*(3,36) = 1.53, *p* = 0.222, S Abd/Ext, *F*(3,36) = 0.723, *p* = 0.555, S Stab, *F*(3,36) = 3.521, *p* = 0.025]. In addition, the two-sample circular test with Bonferroni correction (α/*n* = 0.0125) showed statistically significant difference for: S Stab (Left vs. Right, *p* = 0.001).

### 3.4. Muscle synergies in kinematic reaching across different starting locations

#### 3.4.1. Muscle synergy patterns

In the reaching task, the identified synergy composition was highly similar between starting locations (Figure 4). The six muscle synergy patterns identified in Figure 4 were: elbow flexion (E Flex), elbow extension (E Ext), shoulder adduction and flexion (S Add/Flex), deltoids (Delt), shoulder abduction and extension (S Abd/Ext), and shoulder stabilization (S Stab). Across locations, the E Flex synergy consisted of the activation of BRD, BRCH, and BI muscles, and the E Ext synergy consisted of the activation of TriLat and TriLong, along with some activation of BRD and BRCH. The S Add/Flex synergy contained the activation of AD and PECT with some BI activation in the Distal, Proximal, and Right locations. The Delt synergy consisted of the co-activation of the three deltoid muscles: AD, MD, and PD. This muscle synergy was only identified in the reaching task and not in the isometric force generation task. The S Abd/Ext synergy was composed of the activation of MD, PD, MT, LT, and IN. The S Stab synergy consisted of the activation of UT and IN.

The muscle synergy composition within kinematic reaching task across the four locations was not significantly different [ANOVA; E Flex, *F*(3,36) = 1.15, *p* = 0.343, E Ext, *F*(3,36) = 0.69, *p* = 0.57, Delt, *F*(3,36) = 1.28, *p* = 0.30, S Add/Flex, *F*(3,36) = 0.12, *p* = 0.94, S Abd/Ext, *F*(3,36) = 0.43, *p* = 0.729, S Stab, *F*(3,36) = 1.12, *p* = 0.355]. In addition, two to four out of six muscle synergies between all possible pairs of the arm starting locations were statistically significant (Table 4; *r* > 0.837), meaning that some of the muscle synergies were shared across the starting arm locations. The E Ext, Delt, and S Stab muscle synergy had the least similarity scores with statistical significance. An explanation of the variation of the Delt synergy across starting arm locations may be due to the difference in the muscle weights among the three heads of the deltoid. For example, in Figure 4, the Delt synergy activated at the Proximal location had a much greater AD muscle weight and less MD and PD weight compared to the other three locations. From the cross-validation between different starting arm locations in comparison in the isometric task, the gVAF values of the reconstructed EMGs were close to the synergy identification gVAF threshold value (90%) and within the range of [88.9, 90.88]. The two one-sided test showed that the differences between the reconstructed EMGs and the original EMG falls within the range of 5% in the reaching task, deeming the mean gVAFs between the two groups equivalent (Table 5).

**TABLE 4:** The similarity scores of the muscle synergies (mean ± SD; *n* = 10) for point-to-point reaching between all possible pairs of starting hand locations.

Point-to-point reaching							
Starting hand location comparisons/Muscle synergy		E Flex	E Ext	S Add/Flex	Delt	S Abd/Ext	S Stab
Distal vs. Left	Mean ± SD	0.82 ± 0.24	0.67 ± 0.30	0.89 ± 0.17*	0.64 ± 0.33	0.85 ± 0.16*	0.75 ± 0.31
Distal vs. Right		0.77 ± 0.36	0.78 ± 0.24	0.96 ± 0.04*	0.94 ± 0.07*	0.82 ± 0.31	0.81 ± 0.26
Distal vs. Proximal		0.84 ± 0.28*	0.76 ± 0.31	0.85 ± 0.26*	0.70 ± 0.33	0.77 ± 0.28	0.77 ± 0.28
Left vs. Proximal		0.95 ± 0.09*	0.79 ± 0.25	0.85 ± 0.22*	0.77 ± 0.25	0.84 ± 0.22*	0.91 ± 0.09*
Left vs. Right		0.86 ± 0.24*	0.76 ± 0.26	0.85 ± 0.12*	0.62 ± 0.32	0.78 ± 0.24	0.79 ± 0.28
Right vs. Proximal		0.88 ± 0.24*	0.85 ± 0.18*	0.87 ± 0.15*	0.71 ± 0.33	0.73 ± 0.35	0.80 ± 0.28

*p < 0.0125. E Flex, elbow flexion; E Ext, elbow extension; S Add/Flex, shoulder adduction and flexion; Delt, deltoid synergy; S Abd/Ext, shoulder abduction and extension; and S Stab, shoulder stabilization.

**TABLE 5:** The gVAF of reconstructed EMGs (mean ± SD; *n* = 10) between a pair of starting arm locations in comparison in point-to-point reaching using the cross-validation method.

Starting location/Reconstructed EMGs	Point-to-point reaching			
Distal	**W_D_**×**C_D_**	**W_L_** × **C′_D_**	**W_P_** × **C′_D_**	**W_R_** × **C′_D_**
	91.17 ± 2.01	88.90 ± 2.36***	89.19 ± 2.21***	89.86 ± 2.29***
Left	**W_L_**×**C_L_**	**W_D_** × **C′_L_**	**W_P_** × **C′_L_**	**W_R_** × **C′_L_**
	91.98 ± 1.76	89.57 ± 2.54***	90.23 ± 2.06***	89.14 ± 2.05***
Proximal	**W_P_**×**C_P_**	**W_D_** × **C′_P_**	**W_L_** × **C′_P_**	**W_R_** × **C′_P_**
	91.92 ± 1.72	89.97 ± 2.22***	90.25 ± 2.19***	89.98 ± 2.26**
Right	**W_R_**×**C_R_**	**W_D_** × **C′_R_**	**W_L_** × **C′_R_**	**W_P_** × **C′_R_**
	92.11 ± 1.80	90.88 ± 2.06***	89.22 ± 1.97**	90.23 ± 2.34**

**p < 0.01; ***p < 0.001. W, muscle synergies; C, synergy activation profiles; C′, optimized synergy activation profiles; D, distal; L, left; P, proximal; and R, right.

#### 3.4.2. Synergy activation profiles

**FIGURE 6:**
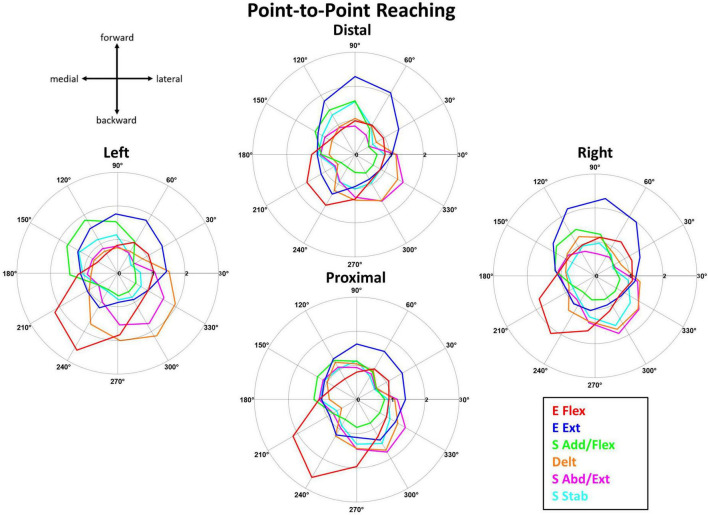
The muscle synergy activation tuning curves showed the averaged (*n* = 10) contribution of each muscle synergy in achieving point-to-point kinematic target matches in one of 12 different directions across the four starting arm locations. The starting arm locations were aligned to the biomechanically forward-backward and lateral-medial directions (see Section “2.4.2. Synergy identification and comparison”). The directionality of the muscle synergies was generally similar across the starting arm locations.

The tuning direction of all but one muscle synergy activation profile was generally consistent across the four starting arm locations during point-to-point reaching. Figure 6 shows that the mean activation (*n* = 10 participants) of the point-to-point reaching muscle synergies across the 12 target directions was plotted at each starting arm location. The tuning directions of E Flex and E Ext activation were opposed, backward-medial and forward-slightly lateral directions, respectively. Also, the tuning directions of S Add/Flex and S Abd/Ext synergy activations were opposing, forward-medial and backward-lateral, respectively. The tuning direction of Delt synergy activation in reaching also opposed the S Add/Flex synergy activation, suggesting that the co-activation of the three deltoid heads was also involved in stabilizing the shoulder joint during shoulder abduction, extension, and external rotation in reaching. The tuning direction of S Stab synergy activation was forward-medial in the Left location and was forward-medial and backward-lateral in the Distal, Proximal, and Right locations. Several muscle synergies were activated per target direction in reaching, suggesting that multiple muscle synergies were required to be activated to complete a point-to-point reach in a certain direction.

The tuning directions within kinematic reaching across the four locations was significantly different (*p* < 0.05) in only the S Abd/Flex synergy [ANOVA; E Flex, *F*(3,36) = 1.59, *p* = 0.208, E Ext, *F*(3,36) = 0.789, *p* = 0.508, S Add/Flex, *F*(3,36) = 1.097, *p* = 0.363, Delt, *F*(3,36) = 0.741, *p* = 0.741, S Abd/Ext, *F*(3,36) = 9.627, *p* < 0.001, S Stab, *F*(3,36) = 0.853, *p* = 0.474]. Two-sample circular test with Bonferroni correction (α/*n* = 0.0125) showed statistically significant differences for: S Abd/Flex (Distal vs. Proximal, *p* = 0.009, Distal vs. Right, *p* < 0.001, Left vs. Proximal, *p* = 0.011, Left vs. Right, *p* < 0.001).

### 3.5. Comparison of muscle synergies between isometric force generation and kinematic reaching

#### 3.5.1. Similarity scores between motor tasks across conditions

Table 6 shows that the composition of more than half of the compared muscle synergies was consistent between the isometric force generation and kinematic reaching at each of the same starting locations of the arm. The E Flex, E Ext, S Add/Flex, SAbd/Ext, and S Stab synergies were determined to be the muscle synergies that were comparable between the two motor tasks. On average, the composition of two, four, two, and three out of the five synergies was statistically similar between isometric contraction and kinematic reaching at the Distal, Left, Proximal, and Right locations, respectively. The least statistically similar muscle synergies were the E Ext synergy and the S Stab synergy across the four locations. The major difference in the composition of the E Ext synergy observed between the two motor tasks was the coupling of biceps with triceps in the isometric force generation task, while triceps were activated alone in the point-to-point reaching. This observation suggested that the elbow stabilization behavior was mainly involved in the isometric force generation task but not in kinematic reaching. In the S Stab synergy, the weights of the co-activation of UT and IN were different between isometric contraction and point-to-point reaching.

**TABLE 6:** The similarity scores of the muscle synergies (mean ± SD; *n* = 10) between the isometric force generation and point-to-point reaching each starting hand location.

Starting hand location/Muscle synergy pair		*W*_*I*−*E Flex*_ × *W*_*K*−*E Flex*_	*W*_*I*−*E Ext*_ × *W*_*K*−*E Ext*_	*W*_*I*−*S Add/Flex*_ × *W*_*K*−*S Add/Flex*_	*W*_*I*−*S Abd/Ext*_ × *W*_*K*−*S Abd/Ext*_	*W*_*I*−*S Stab*_ × *W*_*K*−*S Stab*_
Distal	Mean ± SD	0.73 ± 0.26	0.58 ± 0.20	0.80 ± 0.17*	0.75 ± 0.20*	0.65 ± 0.23
Left		0.79 ± 0.17*	0.63 ± 0.16	0.83 ± 0.10*	0.82 ± 0.14*	0.77 ± 0.16*
Proximal		0.83 ± 0.18*	0.65 ± 0.18	0.67 ± 0.24	0.78 ± 0.18*	0.71 ± 0.24
Right		0.89 ± 0.06*	0.71 ± 0.11	0.83 ± 0.13*	0.80 ± 0.23*	0.68 ± 0.26

*p < 0.05. W_I_, muscle synergies underlying isometric force generation; W_K_, muscle synergies underlying kinematic point-to-point reaching; E Flex, elbow flexion; E Ext, elbow extension; S Add/Flex, shoulder adduction and flexion; S Abd/Ext, shoulder abduction and extension; and S Stab, shoulder stabilization.

The muscle synergy composition between isometric force generation and kinematic reaching across the four locations was not significantly different [ANOVA; E Flex; *F*(3,76) = 1.34, *p* = 0.268, E Ext; *F*(3,76) = 0.66, *p* = 0.58, S Add/Flex; *F*(3,76) = 1.69, *p* = 0.175, S Abd/Ext; *F*(3,76) = 0.22, *p* = 0.885, S Stab; *F*(3,76) = 0.6, *p* = 0.62]. The Delt synergy was not included in the test because it is a point-to-point reaching specific synergy.

#### 3.5.2. Number of synergies recruited per target direction

**FIGURE 7:**
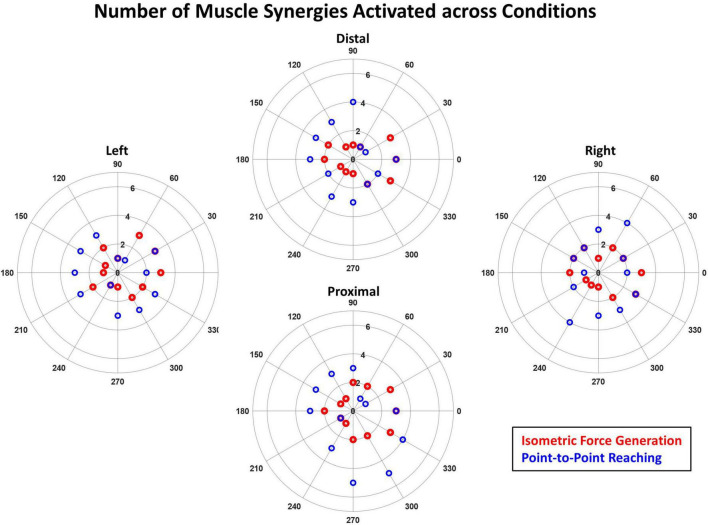
The number of muscle synergies significantly activated across the 12 target directions in the isometric force generation (red) and point-to-point reaching tasks (blue) based on the mean muscle synergy activation across participants. The purple circles indicate the case when the number of significantly activated synergies is the same for both isometric and kinematic reaching in a given direction. The magnitude of the mean synergy activation greater than the activation threshold (see Section “2.4.2. Synergy identification and comparison”) was counted as significant. The synergy activation magnitude was typically higher in the point-to-point reaching task than in isometric force generation across the 12 targets at each starting arm location.

The number of muscle synergies activated during kinematic reaching was typically one greater than that of isometric force generation in the horizontal plane, suggesting that reaching involved higher task complexity (Figure 7). The activation thresholds of isometric and kinematic tasks (*t_ISO* and *t_KINEMATIC*) determined, based on the calculation method described in Section “2.4.2. Synergy identification and comparison”, at each of the four starting locations were: at Distal, *t_ISO* = 1.28, *t_KINEMATIC* = 1.04; at Left, *t_ISO* = 1.31, *t_KINEMATIC* = 1.09; at Proximal, *t_ISO* = 1.29, *t_KINEMATIC* = 1.10; and at Right, *t_ISO* = 1.29, *t_KINEMATIC* = 1.14. The ranges of the number of muscle synergies activated across the different starting arm locations were: at Distal, *n_ISO* = 1–3 synergies, *n_KINEMATIC* = 1–4 synergies; at Left, *n_ISO* = 1–3 synergies, *n_KINEMATIC* = 1–3 synergies; at Proximal, *n_ISO* = 1–3 synergies, *n_KINEMATIC* = 1–5 synergies; and at Right, *n_ISO* = 1–3 synergies, *n_KINEMATIC* = 1–4 synergies. Across the locations, except for the Left location, the point-to-point reaching required a higher range of the number of muscle synergies compared to that of the isometric force generation.

## 4. Discussion

This study aimed to examine to what extent muscle synergies of isometric force generation and point-to-point reaching were shared in motor control of the human upper extremity. We found that across the four different arm positions, the number and composition of muscle synergies were comparable between isometric force target matches and kinematic reaching despite potential differences in afferent feedback. Typically, five and six muscle synergies underlaid isometric force generation and point-to-point reaching each arm location, respectively. Moreover, although the EMG profiles were distinct between the two motor task trials (Figure 2), when we compared the intermuscular coordination patterns identified from the isometric and kinematic reaching tasks, common muscle synergies were identified between both conditions. Also, the tuning direction in the majority of synergy activation profiles underlying each motor task were not statistically different between the four different arm locations. Two to four out of five muscle synergies of isometric contraction were found to be in common with reaching, depending on the arm location in the workspace, and one to three out of five muscle synergies of the isometric task were task-specific. To date, our study was the first attempt to compare the synchronous intermuscular coordination of the isometric force control and kinematic reaching across the arm workspace in the human upper extremity.

The generalizability of muscle synergies between the isometric and kinematic reaches and across different arm locations within the arm workspace supported the concept of muscle synergies as a neuromuscular control mechanism for motor coordination. The CNS and the musculoskeletal system orchestrate the activation of muscles in groups, instead of controlling them individually, by activating a small number of consistent muscle co-activation patterns to produce both isometric and reaching tasks. Similar to previous studies that examined the isometric (Roh et al., 2012) and the reaching tasks (d’Avella et al., 2006, 2008; Muceli et al., 2014) individually, our results (Figures 3A, B) showed that a limited number of muscle synergies were able to represent the EMG of 13 different arm and back muscles recorded during the isometric and reaching tasks, respectively. In addition, our findings in Table 2 found that most muscle synergies were conserved across the human arm workspace within the isometric force generation task. Our findings in Table 4 indicated that two to four out of six muscle synergies were conserved across the starting arm locations in the reaching task. When comparing the sets of muscle synergies per motor task using the cross-validation method, the reconstructed EMGs from the muscle synergies and activation profiles between all possible combinations of starting arm locations in comparison resulted in high gVAF values and was deemed similar to the mean gVAF value of the original EMG at each starting arm location (Tables 3, 5). Through our findings, the conservation of muscle synergies within the arm workspace of each motor task resembles the findings of previous studies in isometric (Roh et al., 2012) and reaching tasks (d’Avella et al., 2006). Our study further compared the muscle synergies between the isometric and reaching tasks through similar experimental conditions and target-matching tasks (Table 6) and found that more than half of the compared muscle synergies were shared between the motor tasks across starting arm locations even though the sensory afferent information would be very different between isometric and kinematic conditions. Our results were congruent with the findings of a lower extremity study, where three to five out of five to six muscle synergies were shared in both perturbed walking and balance or reactive balance and unperturbed walking (Chvatal and Ting, 2013). The shared muscle synergy patterns seen in the comparison between motor tasks support the hypothesis that the CNS coordinates movement through the limited number of muscle co-activation patterns, often redundant, in the upper or lower extremities.

The differences in the composition of the muscle synergies between the isometric and kinematic reaching tasks indicated the involvement of task-specific and posture-specific biomechanical constraints in the motor coordination of the two tasks. In previous comparisons of movement tasks (d’Avella and Bizzi, 2005; Chvatal and Ting, 2013), differences in motor actions such as human walking and balancing or frog jumping, swimming, and walking resulted in some distinctive muscle synergies observed in each respective task. In the same way, Figure 4 shows examples of task-specific muscle synergies. For example, the composition of the E Flex and E Ext synergies between the isometric and kinematic reaching tasks was different in the way that the BI muscle was activated with E Ext and E Flex synergies in the isometric and reaching tasks, respectively. The co-activation of the BI with the E Ext synergy can best be explained as an elbow stabilization mechanism when the arm is extended. The representative EMG signals of isometric and kinematic reaching tasks (Figure 2) also reflected this co-contraction in the isometric task, which was not present in the kinematic reaching, and was aligned with the results of the synergy analysis. Another example of the difference in the muscle synergy composition was the addition of the Delt synergy in the kinematic reaching task, which seems to act as a shoulder stabilizer during the shoulder abduction, extension, and external rotation movement. These muscle synergies included both co-contracting antagonist muscles around the shoulder and elbow joints, which might allow the enhanced accuracy of the target match in both motor tasks (Gribble et al., 2003). Previous studies of isometric force generation have also shown different muscle synergy structures; E Flex was the co-activation of the BRD and BI muscles, and E Ext was the co-activation of TriLat and TriLong muscles (Roh et al., 2012, 2013, 2015). A noticeable difference between these previous studies and our current study was the participant’s arm positioning. In our previous study (Roh et al., 2012), the participant’s hand was positioned in front of the shoulder at 60% of arm length, while in our current study, the participant’s shoulder was abducted to 80° to hold onto the grasping handle that was aligned with the front of their chest. So, we reason that the minor differences in the muscle synergy composition observed in both motor tasks may be due to task-specific and arm-posture-specific mechanisms.

The differences between the tuning directionality of the isometric versus reaching muscle synergy activation profiles and the activation of a higher number of synergies to perform the target match in kinematic reaching, compared to isometric force generation, suggested that the kinematic reaching task was more complex compared to isometric force generation. For example, Figures 5, 6 show the difference in the tuning direction of the E Ext synergy activation profile between the two tasks. The E Ext was activated in the forward-lateral and forward directions for the isometric and kinematic reaching tasks, respectively. The kinematic reaching task also had a higher number of muscle synergies activated across the 12 target directions (Figure 7), suggesting that multiple muscle synergies were involved in moving and stabilizing the shoulder and elbow joints during the point-to-point reaching in a certain direction. The activation profiles of the muscle synergies underlying kinematic reaching also resembled what has been observed in a similar previous study (Muceli et al., 2010) where the same target-reaching in the horizontal plane has been examined. Out of the four muscle synergies identified in the previous study, the elbow extension and elbow flexion synergies were activated approximately in the forward and backward directions, respectively. The study also reported that multiple synergies were activated simultaneously to make a point-to-point reach toward each target direction, as observed in our current study. The more complex muscle synergy coordination in the reaching task may explain why chronic stroke survivors face more difficulty in performing arm reaching as compared to isometric force generation. This finding also implied that the isometric force exercises could be more accessible to stroke survivors since the task may have a simpler motor mechanism and can be safe for post-stroke individuals who suffer from muscle weakness or muscle spasticity.

Overall, our findings support the neural basis of muscle synergies since only a small number of muscle synergies represented each motor task, and shared muscle synergies were found between the two motor tasks. Although it is difficult to nullify the argument for or against muscle synergies as a neural mechanism of motor coordination (Tresch and Jarc, 2009; Hong et al., 2021), muscle synergy analysis has been used to understand the characteristics of motor control and impairments as well as to design novel therapeutic methods. The argument against muscle synergies has been made by observing individual muscle activations and their contribution to a specific task, especially in cadaveric and biomechanical simulation studies. The previous study done by Valero-Cuevas and his colleagues observed the activation of each muscle during an isometric index finger movement task and its EMG variability throughout individual trials and participants (Valero-Cuevas et al., 2009). The study concluded that the muscles involved in performing the action did not resemble muscle synergies since more task-irrelevant variability was observed across participants. Kutch and his colleagues also explored muscle activity during the fingertip isometric force generation task and concluded that the muscles were independently controlled by the CNS, the opposite of the muscle synergy theory (Kutch et al., 2008). Kutch and Valero-Cuevas then used cadavers and computational models to explore possible muscle synergies through biomechanics without the involvement of the CNS to suggest that constraints from the musculoskeletal system may influence the muscle synergy outcome (Kutch and Valero-Cuevas, 2012). On the contrary, de Rugy and his colleagues argued that motor coordination was more habitual in nature by conducting a series of experiments that involved five different wrist muscles across conditions and postures (de Rugy et al., 2012). They found that robust muscle co-activation patterns were recruited to overcome the different conditions applied, suggesting that the CNS does not seem to optimize the control of individual muscles but instead coordinates groups of muscles to complete a task. However, to prove or disprove the concept of muscle synergies, further research into the neuronal motor pathways needs to be done to validate if muscle synergies were utilized as a mechanism for motor coordination of the musculoskeletal system.

There are some limitations in the methodology of our study. One limitation of this study was that the full human arm workspace was not fully represented in the horizontal plane across the four starting arm locations, due to the range-of-movement constraint in the devices this study used. To compare the starting arm locations across the human arm workspace, the entire reachable area within the horizontal plane of the KINARM Exoskeleton was measured. The workspace of the exoskeleton was limited in the far medial, far lateral, and far backward direction due to the limitations of the shoulder and elbow robot arm joint rotation and the fixed rectangular display that was smaller than the maximum human reaching range. Thus, the participants were not able to see targets located outside of the visual field, limited by the robot mechanics. Another limitation of this study was the constraint of the motor tasks to only the horizontal plane. The KINARM Exoskeleton was chosen as the equipment to study the point-to-point reaching motor tasks as it is a well-known device in the field of movement rehabilitation research since it allows the user to easily perform reaching movements and achieve task-specific goals. With a better representation of the arm workspace in different planes of movement, we can further assess the possible differences in the muscle synergies at different arm locations. The sample size of the participants in this study is another limitation, although the sample size is comparable to previous upper extremity muscle synergy studies (Roh et al., 2012; Muceli et al., 2010; Borzelli et al., 2013; Coscia et al., 2014) and the degree-of-freedom of the experimental conditions (i.e., 12 force target directions) was smaller than the number of participants. In addition, a potential limitation of this study was that the duration of the isometric force generation tasks and reaching tasks varied based on the performance of each participant. A recent isometric force generation study (Seo et al., 2022) has shown that the muscle synergy composition does not change across the early ramp, middle ramp, late ramp, and hold period in healthy participants. The early ramp and the holding period displayed the conservation of muscle synergies which suggests that the possible process of joint stabilization and muscle fatigue in the hold period does not influence the muscle synergy composition.

The generalizability of muscle synergies in the upper extremity may be an indicator that the CNS controls muscle activation in the arm with some shared neural mechanisms. The muscle synergies similar between isometric force generation and kinematic reaching provide a scientific rationale that isometric exercises could be a helpful alternative training method for patients with motor impairment to eventually gain kinematic reaching. As a next step, we plan to further investigate the generalizability of isometric and kinematic reaching in stroke and identify the degree to which possible abnormal muscle synergies are shared between the two motor tasks. We also plan to examine whether the activation of abnormal synergies of the isometric task may correlate with the impaired performance of kinematic reaching. Since stroke survivors have varying levels and types of cortical injuries, we also plan to assess and compare the muscle synergies of stroke survivors with mild, moderate, and severe motor impairments. Overall, these studies can contribute to uncovering the abnormal changes in the potentially shared neural pathways that could contribute to motor dysfunction under varying biomechanical conditions. The resultant scientific knowledge can be translated into creating potentially better and more effective rehabilitation strategies after stroke.

## Data availability statement

The original contributions presented in the study are included in the article/Supplementary material, further inquiries can be directed to the corresponding author.

## Ethics statement

The studies involving human participants were reviewed and approved by Institutional Review Board of the University of Houston. The patients/participants provided their written informed consent to participate in this study.

## Author contributions

KP collected the data, completed data analysis and interpretation, and manuscript writing. JR and MP-J designed the study, collected the data, assisted in data analysis and interpretation, and wrote the manuscript. All authors read and approved the final manuscript.
