# Supplementary material for: Generalizability of muscle synergies in isometric force generation versus point-to-point reaching in the human upper extremity workspace
Source: Front Hum Neurosci. 2023 Jul 17;17:1144860. doi: 10.3389/fnhum.2023.1144860 (PMC10387555; doi:10.3389/fnhum.2023.1144860)
Supplement: Supplementary file 1 [file Image_1.pdf]

Supplementary Material

1 Supplementary Information

1.1 Supplementary Figures

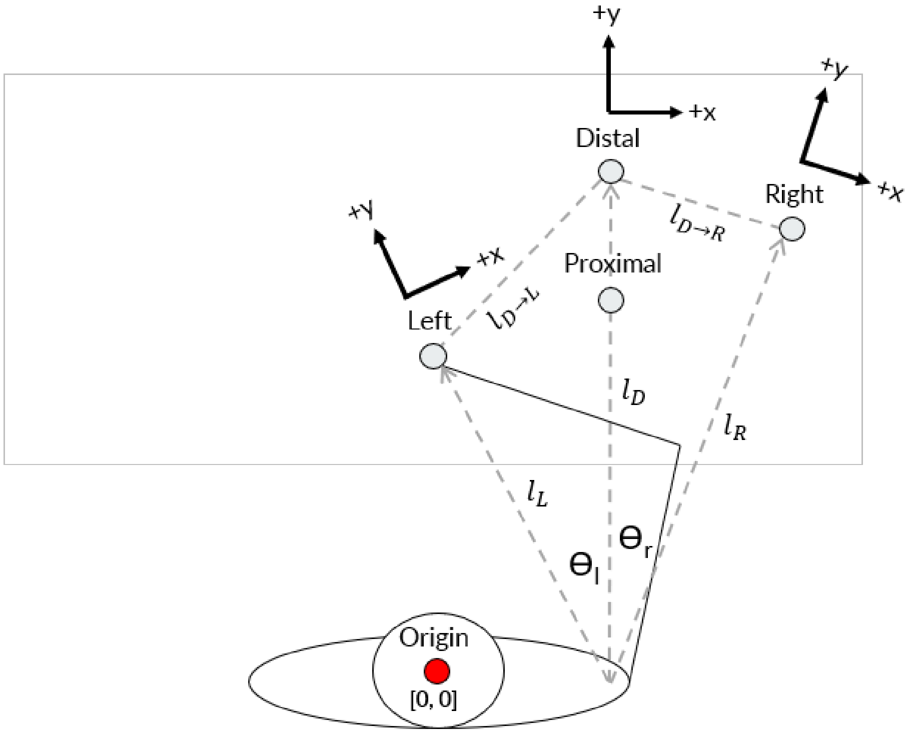

Supplementary Figure 1. The human arm workspace with the defined local coordinate system at different starting arm locations. This local coordinate system was used to compare the synergy activation tuning profiles after aligning the biomechanically forward and lateral directions across the four starting locations. The local coordinate system was defined by setting the y-axis as the vector direction from the shoulder joint to the grasping handle at each starting arm location. Θ_l and Θ_r were calculated to align the coordinates of Left and Right locations to the other starting locations. To shift the Left and Right starting locations to the same local coordinate system as the Distal and Right starting locations, the angle of difference between the Distal and Left vector (Θ_l) and between the Distal and Right vector (Θ_r) were calculated. First, the average distance from the acromion (shoulder joint) to the grasping handle at the Distal ($\boldsymbol{l_D}$), Left ($\boldsymbol{l_L}$), and Right ($\boldsymbol{l_R}$) locations were measured. The distances between the Distal and Left location ($\boldsymbol{l_{D\to L}}$) and the Distal and Right location ($\boldsymbol{l_{D\to R}}$) were calculated using the average global coordinates recorded from the KINARM Exoskeleton. The average lengths (n = 10) that were measured from all participants were: $\boldsymbol{l_D}$ = 50 ± 1.8 cm, $\boldsymbol{l_L}$ = 41 ± 1.6 cm, $\boldsymbol{l_R}$ = 51 ± 1.7 cm, $\boldsymbol{l_{D\to L}}$ = 23.7 ± 0.37 cm, $\boldsymbol{l_{D\to R}}$ = = 19.8 ± 0.1 cm. The three lengths for the left and right start locations were then used to solve Θ_l and Θ_r through the Law of Cosines. The calculated angles were: Θ_l = 28° and Θ_r = 22.4°. (see section 2.4.2). +x, lateral; +y, forward.
